# Early Autism Intervention Components Deliverable by Non-specialists in Low- and Middle-Income Countries: A Scoping Review

**DOI:** 10.3389/fpsyt.2022.914750

**Published:** 2022-06-29

**Authors:** Lavangi Naithani, Caitlin Goldie, Abhipreet Kaur, Charlotte Butter, Shweta Lakhera, Kathy Leadbitter, Gauri Divan

**Affiliations:** ^1^Sangath, Goa, India; ^2^Division of Neuroscience and Experimental Psychology, The University of Manchester, Manchester, United Kingdom

**Keywords:** low- and lower-middle-income countries, non-specialist delivery, autism intervention components, autism spectrum disorder (ASD), low resource setting

## Abstract

**Introduction:**

The past decade has seen key advances in early intervention for autistic children in high-income countries, with most evidence based on specialist delivery of interventions. The care gap seen in low- and middle-income countries (LMIC) remains close to 100%. A key challenge in addressing this care gap concerns the paucity of specialists available to deliver services. Task-sharing provides an important potential solution; there is a need to identify interventions that are suitable for scaled-up delivery through task-sharing in low-resourced settings. We aimed to conduct a scoping review to identify studies which reported autism intervention delivered by non-specialists within LMIC and, using established frameworks, specify intervention components with evidence of successful non-specialist delivery.

**Methods:**

A scoping literature search, conducted within four databases, generated 2,535 articles. Duplicates were removed, followed by screening of titles and abstracts, with 10% double-rated for reliability. 50 full text articles were then screened independently by two raters. Articles were included if studies: (a) were conducted in LMIC; (b) included samples of autistic children (age < 10); (c) evaluated psycho-social interventions delivered by non-specialists; (d) reported child outcomes; and (e) were peer-reviewed full-texts in English. Two established frameworks – @Practicewise and NDBI-Fi framework - were then used to ascertain the commonly delivered components of these interventions.

**Results:**

Two studies met the inclusion criteria. Both studies evaluated parent-mediated interventions delivered by non-specialists in South Asia. Through the two frameworks, we identified elements and techniques that had been delivered successfully by non-specialists.

**Conclusion:**

There is evidence from two acceptability and feasibility trials that non-specialists can be trained to deliver some intervention elements and techniques within parent-mediated interventions, with good fidelity and acceptability and evidence of effectiveness. The review points up the lack of a widespread evidence base in this area and need for further research in low resourced settings, including well-powered trials and mechanistic analyses to identify active ingredients. A focus on the pre-requisites for non-specialist delivery is critical to reduce inequity and provide universal health coverage within resource-constrained health systems.

## Introduction

Autism Spectrum Disorder, more commonly referred to as autism, is a lifelong neurodevelopmental disability characterized by impairments in social communication, restricted, repetitive interests and behaviors, and differences in sensory processing, with the presence of co-occurring intellectual disability in up to a third of individuals (1). A review of global prevalence reported 1–1.8% (2), with similar findings in studies from low- and middle-income countries (LMIC), with prevalence rates of 1.04 and 1.1% in Sri Lanka and India, respectively (3, 4). In the majority of LMIC, there exist a number of obstacles to accessing care which include the low awareness of the condition and the lack of access to detection and treatment services. Many LMIC have little or no policy implementation and underfunded health care systems for child and adolescent services (5, 6). This results in a significant “detection” gap in the identification of autism in young children, as well as a “treatment gap” with a lack of evidence-based interventions for the children who do receive a diagnosis.

Low- and middle-income countries have a significant proportion of their populations in the younger demographic age groups. This, coupled with large detection and treatment gaps, makes the need to identify interventions that can be scaled up a critical component of the right to care for young children with autism but also for the attainment of the United Nations Sustainable Development Goal 3. This goal aims to ensure the healthy lives and the promotion of health for all at all ages (7). While some LMIC settings do have services, these are predominantly focused in metropolitan areas and remain impacted by the scarcity of specialists with expertise in child development, including developmental pediatricians, child psychiatrists, speech and language therapists or occupational therapists (6). There is a need for radical innovations to help scale up services in these low resource contexts. Across many areas of health, including within treatment for mental health conditions like depression, innovations have used the process of “task-sharing” to address this dearth of specialist care (8). This approach aims to systematically skill up a non-specialist or para-professional with intervention-specific knowledge and skills, such that they are competent to facilitate the delivery of an evidence-based intervention in the community. Task-sharing is usually supported by a strong supervisory framework: the non-specialist has focused knowledge in a specific intervention or area of care, but more expert advice or care is readily available in the event of complex cases or scenarios. This approach allows an intervention to be delivered by a non-specialist, with the mobility to reach out to families in the community. Thus task-sharing with accessible non-specialist community-based facilitators overcomes barriers such as the limitation of specialist services, the expenses of such services, and the geographical distance to services.

The aim of this study was to conduct a scoping review and identify interventions for young children with autism that have evidence of feasibility, acceptability and effectiveness when delivered by non-specialists in LMIC. We adopted a narrow definition of “non-specialist” informed by the availability of personnel for task sharing to deliver care to children and families in LMIC settings (9), rather than the workforce available within low-resourced settings in high-income countries. These providers are typically frontline community health workers (10) who are not specialists in child development, autism, or mental health but who are already working on the ground within local communities. Examples include the Accredited Social Health Activists working in Delhi (11), Female Community Health Volunteers in Nepal (12), and Community Health Volunteers working in Kenya (13). Other potential providers include community volunteers or parent/disability “champions”. Our definition of specialist providers included those specialist professionals largely unavailable within LMIC: pediatricians, psychiatrists, nurse practitioners, psychologists, speech and language therapists, occupational therapists, physical therapists, and students/trainees of these professions. We focused on interventions delivered within a family context rather than school-based or teacher-delivered interventions, which tend to center around educational outcomes. In addition, we sought to address inconsistency in the definition around non-specialist delivery found within the literature. We differentiated between non-specialist-*mediated* interventions, and non-specialist *delivery*, the latter being the focus of our search. The former includes a broad range of approaches in which a practitioner delivers the intervention to a dyad (e.g., parent-child, sibling-child, peer-child) and the agent of mediation between the practitioner and autistic child (e.g., parent, sibling, peer) is considered a non-specialist. Some reviews (14) identified the parent receiving the intervention as the non-specialist and included all studies of parent-mediated interventions even where the intervention was delivered to the parent by a specialist. To meet our definition, parent-mediated interventions were included only when delivery to the parent-child dyad was by a non-specialist. This was a purposeful distinction to ensure that the focus was on interventions deliverable to families by non-specialists in LMIC. We also excluded interventions which were delivered by both specialists and non-specialists in tandem (15).

Following the identification of studies meeting our criteria, we sought to characterize the common elements (defined as a therapeutic activity or strategy) and techniques (defined as skills used during a session to deliver an element) of interventions that have been delivered by non-specialists under supervision and evaluated in LMIC countries. We were guided by two published frameworks: (1) the model proposed by Chorpita et al. (16) for delineating common ingredients of interventions for young children and adolescents which allows for the design of scalable evidenced modular interventions; (2) the framework developed by Frost and colleagues (17) through an expert-led Delphi approach, which aims to characterize the common elements and components of naturalistic developmental behavioral interventions (NBDIs) for autism.

## Methods

We followed the five-step framework for scoping reviews suggested by Levac and colleagues (18). We firstly defined the research question and then identified the relevant studies based on predetermined inclusion and exclusion criterion. We then charted and summarized the data. We describe these steps in detail below.

An OVID literature search was conducted within four databases (APA PsychInfo, APA Psycharticles, Ovid Medline + Epub Ahead of Print, Embase) to identify relevant studies. Search terms are shown in Table 1. This search identified 1,721 articles after 808 duplicates were identified and removed within OVID.

**TABLE 1:** OVID search terms.

Category	Search terms	Search fields
Condition	autis*, pervasive	In title
Age-group	Child*	Anywhere in article
Article Type	Primary research	
LMIC context, global health or culture	LMIC*, LAMIC*, low resource*, low-income, limited resource*, middle-income, glob*, cultur*	Anywhere in article
Delivery agent	Paraprofessional*, para-professional*, semi-professional*, lay, frontline, front-line, non-specialist*, task*sharing, aide*, assistant*	Anywhere in article
Focus on intervention	care, treatment*, therap*, intervention*, train*, service*, group*	In abstract, keywords or title

*The asterix is used as a standard method of describing search terms in a review.*

**FIGURE 1:**
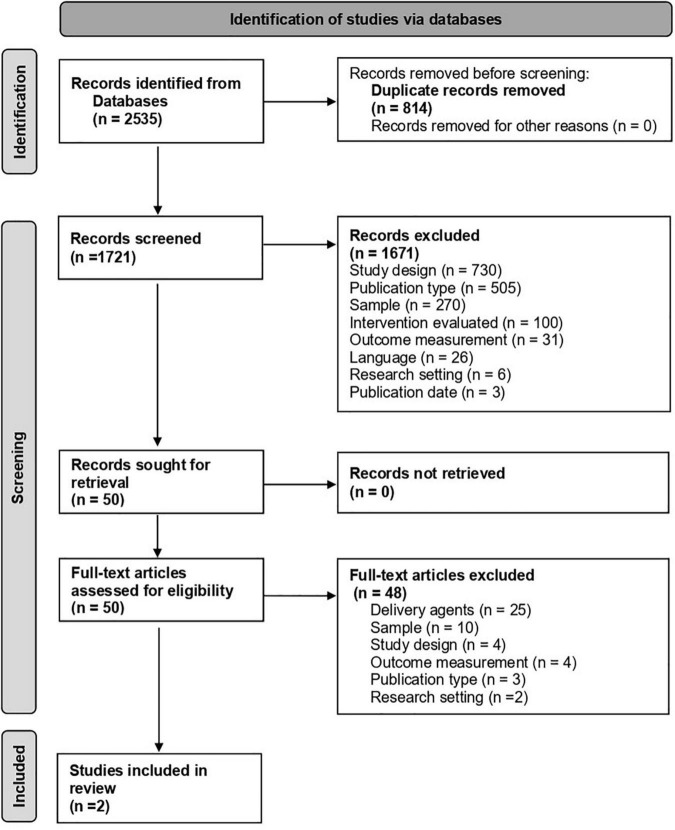
PRISMA flow diagram of the study selction process.

These articles were imported into Rayyan (19), a free web application designed for collaborative reviews. Rayyan identified 24 potential duplicates. These were checked manually and 6 were deleted. To establish inter-rater reliability prior to the title and abstract screen, 10% (173) were screened by all five reviewers, maintaining blind to each other’s inclusion/exclusion decisions. Agreement was reached for 130 articles (75%). Disagreements and uncertainties were resolved through discussion and clarification of terminologies and inclusion/exclusion criteria with support from two senior authors, until consensus was reached. Of these articles, 166 were excluded and 7 included for full-text review. The remaining 1,542 articles were divided up between five raters (AK, SL, LN, CB, and CG). All raters had at least a relevant undergraduate degree (CB, CG) or Masters degrees or equivalent (LN, AK, and SL) and were working within autism intervention research projects. The raters screened the title and abstracts, applying the inclusion/exclusion criteria shown in Table 2. This resulted in 1,499 articles being excluded and 43 articles put forward for a full text review. Together with the articles used for inter-rater reliability, a total of 1,671 articles were excluded and 50 articles (7 from reliability screen, plus 43 from individual screen) were put forward for full text review. The full breakdown of exclusion reasons across both stages can be found in the PRISMA (Figure 1).

**TABLE 2:** Inclusion/exclusion criteria.

Domain	Inclusion criteria	Exclusion criteria
Language	In English language	Not in English language
Publication type	Published and peer-reviewed empirical articles	Reviews Chapters from books Correction notices Study protocols Guidelines and clinical protocols Unpublished dissertations/theses
Publication date	Published 2006–2021	Published before 2006
Research setting	Low or middle income country	High income country
Sample	Sample (recipients of intervention) includes children with a diagnosis of autism or parents/caregivers of young children with a diagnosis of autism Sample includes only or mainly children under 10 years Sample > 10 participants	Sample (recipients of intervention) is medical or educational professionals Sample contains only adults or only/mainly children above the age 10 Sample < 10 participants
Study design	Feasibility or pilot randomized controlled trials Large randomized controlled trials Pre- and post-intervention design	Epidemiological studies Cross-sectional studies Surveys Purely qualitative studies Case studies or case series Descriptive
Outcome measurement	Studies that include measurement of child outcomes relating to core features of autism (e.g., social communication, interaction, play and behavior)	Studies which report feasibility/acceptability but not outcomes Studies which report only parent/caregiver outcomes School based interventions focused on curriculum outcomes
Intervention evaluated	Psycho-social intervention targeting core features of autism	Interventions solely targeting non-core features of autism, e.g., sleep, motor control Technological interventions, e.g., robots, computer avatars etc. Pharmacological interventions Biomedical treatments e.g., diets, hyperbaric O2 etc.
Delivery agents	Intervention delivered by non-specialists (see definition)	Intervention delivered completely or partly by specialists, trainees or students of professional courses or teachers Intervention is self-directed Child peer-delivery or -mediation

The full-text review of the 50 articles was carried out independently by two raters from the pool of five raters, applying the same inclusion/exclusion criteria. There were 6 conflicts at this stage and these were resolved through discussion with the wider coding team. This led to the exclusion of 48 articles and the inclusion of 2 articles.

The next stage involved reviewing 18 articles recommended through key informants and bibliographic searches. These were divided between the 5 raters who, again, applied the same inclusion/exclusion criteria. These reviews produced two articles, which were the same articles that resulted from the search.

### Common Components

Chorpita and Daleiden (20) developed a framework for identifying the components of psychosocial interventions for mental health conditions in children and adolescents which had demonstrated “favorable” results in effectiveness evaluations. The framework is descriptive in nature and does not seek to pinpoint the critical or “active” elements of these interventions. These identified elements have been collated into a coding manual called @PracticeWise (21). We examined the elements and techniques mapped in the @Practicewise common elements framework (20), and calibrated these broad definitions to encompass the needs of autism interventions along with the common components defined for the NBDI fidelity measure (17). LN and GD mapped the elements and techniques by reading the description of the intervention in the publication and manuals, plus drawing upon their professional understanding of the interventional approaches. We defined *elements* as a therapeutic activity or strategy and *techniques* as a skill that a therapist uses before or during the session to deliver an element (16).

## Results

Two publications met our full search criteria, see Figure 1. Both these papers reported well-designed evaluations of interventions for young autistic children delivered in LMICs by non-specialists with no previous training in child development. Characteristics of the studies, interventions, and delivery agents are summarized in Table 3. Rahman et al. (22) trialed a parent-mediated social communication-focused intervention (Parent mediated intervention for Autism Spectrum Disorders in South Asia: PASS) delivered by non-specialist health workers, at home or in a clinic, in Rawalpindi, Pakistan and Goa, India. The intervention was an adaptation of the UK Pre-school Autism Communication Therapy (PACT), which was modified for delivery by non-specialists working within a competency-based training and supervision cascade led by specialists (23). The “non-specialists” were bachelor level graduates with no prior exposure to child development or autism. Precisely as in the original PACT intervention, they used video-feedback methods to enhance the parents’ sensitivity to their children’s non-verbal and verbal communication and refine the relevance of their responses to their child (known as parental “synchrony”). Parents were also encouraged, in later stages of the intervention process and where appropriate, to introduce action routines, repetitive language and pauses with the aim of aiding the child’s communication development. Implementation of strategies was encouraged between therapy sessions. Children with a diagnosis of autism aged 2–9 years were randomly assigned (1:1) to the PASS intervention (*n* = 32) or the treatment as usual (TAU) condition (*n* = 33), with controls for age (<6 or >6), functional impairment and treatment center location (Rawalpindi or Goa). The outcome measures for this initial effectiveness trial included parental synchrony and child communicative initiations (measured on the Dyadic Communication Measure for Autism) (24), parent reports of child adaptation and language use (measured on the Vineland Adaptive Behavior Scale (25), and the McArthur Communicative Development Inventory (26), respectively), the verbal and non-verbal aspects of social communication (measured on the Communication and Symbolic Behavior Scales Developmental Profile) (27) and maternal depressive symptoms (measured using a Patient Health Questionnaire-9) (28). The intervention proved feasible and acceptable for participants (only 9% of participants were lost to follow up). Additionally, and importantly, the intervention was delivered by the non-specialists with high and sustained therapist fidelity to the intervention manual (89% of intervention sessions meeting the pre-defined threshold for acceptable fidelity). For outcomes, in comparison to TAU, PASS increased parental synchrony and the initiation of communication by the children in the dyad, but child-specific outcomes beyond dyad were not affected. Other results are reported in Table 3.

**TABLE 3:** Key characteristics of studies identified by the scoping review.

	Rahman et al. (22)	Divan et al. (29)
**Study characteristics**		
Location of research	South Asia (Rawalpindi, Pakistan and Goa, India)	South Asia (Kolhapur, India)
Design	Single-blind effectiveness RCT	Single-blind effectiveness RCT
Name of intervention	PASS with treatment as usual	PASS-Plus with treatment as usual
Comparison	Treatment as usual (educational provision, some speech and language therapy)	Treatment as usual (educational provision, some speech and language therapy)
Follow-up period	8 months	9 months
Child diagnosis	Professional diagnosis of ASD	Professional diagnosis of ASD
Age of children	2–9 years	2–9 years
Number of participants	65	40
**Intervention characteristics**		
Supervision	Group and peer supported	Group and peer supported
Intervention Approach	Interventionist to Parent	Interventionist to Parent
Length of intervention	Fortnightly, 12 sessions over 6 months	Fortnightly, 12 sessions over 6 months
Treatment target	Parent-child synchrony and child initiations	Autism symptom severity, parent-child synchrony, shared attention and child initiations
Intervention Techniques	Video-feedback of parent-child play interactions and psychoeducation (see Tables 4, 5)	Video-feedback of parent-child play interactions, psychoeducation, and separate section addressing commonly co-occurring conditions, with discussions, collaborative goal setting and supportive illustrated handouts to support low literacy (Tables 4, 5).
**Study results**		
Feasibility, acceptability and fidelity	- Good feasibility for delivery within both Goa and Rawalpindi contexts and in children up to age 9, including those with high support needs - Good acceptability - Low attrition- 10% was allowed for; actual rate was 9% - High therapist fidelity to the intervention manual sustained throughout the trial	- High feasibility for delivering the intervention in a rural setting - Good acceptability - Attrition/attendance: 89% of participants received at least 3 sessions and 68% received the maximum 12 - High therapist fidelity to the core PASS intervention manual; lower fidelity for the Plus modules in terms of lay health workers tailoring to families’ needs
Parent-child dyadic communication	Dyadic Communication Measure for Autism (DCMA) (24) Parental synchrony: Adjusted Mean Difference (AMD) 0.25; 95% CI 0.14 to 0.36; Effect Size (ES) 1.61 Proportion of child communication initiations with parent: AMD 0.15; 95% CI 0.04 to 0.26; ES 0.99 Child-parent mutual shared attention: AMD −0.16; 95% CI –0.26 to –0.05; ES −0.70	Dyadic Communication Measure for Autism (DCMA) (24) Parental synchrony: AMD 0.35; 95% CI 0.18 to 0.52; ES 3.97 Proportion of child communication initiations with parent: AMD 0.17; 95% CI 0.03 to 0.32; ES 1.02 Child-parent mutual shared attention: AMD 0.10; 95% CI −0.07 to 0.27; ES 0.5 Co-morbid symptoms: AMD −9.0; 95% CI −24.26 to 6.26; ES 0.32
Parental mental health	Patient Health Questionnaire-9 (28) AMD 0⋅95; 95% CI –1⋅38 to 3⋅27; ES 0.27	Patient Health Questionnaire-9 (28) AMD −4.55; 95% CI −8.52 to −0.58; ES 0.76
Child adaptive behavior	Vineland Adaptive Behavior Scale (VABS) (25) Adaptive Behavior Composite: AMD –0⋅93; 95% CI –3⋅53 to 1⋅68) ES –0.08	Vineland Adaptive Behavior Scale (VABS) (25) Adaptive Behavior Composite: AMD 0.67; 95% CI −3.80 to 5.15; ES 0.06
Child communication and language	McArthur Communicative Development Inventory (26) Receptive subscale: AMD 0⋅19; 95% CI –0⋅56 to 0⋅94; ES 0⋅07 Expressive subscale: AMD 0⋅03; 95% CI –0⋅46 to 0⋅52; ES 0⋅01 Communication and Symbolic Behavior Scales Developmental Profile (27) Total score: AMD –0⋅68; 95% CI –8⋅79 to 7⋅42; ES –0⋅02	N/A
Child behavior	N/A	Developmental Behavior Checklist (DBC) (30) Total score: AMD −9.00; 95% CI −24.26 to 6.26; ES 0.32
Child autism symptoms	N/A	Brief Observation of Social Communication Change (BOSCC) (31) AMD −2.42; 95% CI −7.75 to 2.92; ES 0.22

During the PASS trial it became apparent that co-occurring non-autistic difficulties were often un-treated due to the lack of other generic child development and health services in the community, and that these co-occurring problems such as those associated with sleep, toilet training, parental wellbeing, and dietary restrictions, could hinder access to the core PASS technique. A number of additional modules were therefore devised for incorporation when necessary, on the basis of the reasonable effectiveness evidence for each co-occurring difficulty but simplified such that they could be within the competence of non-specialists. The implementation of these was based on a clinical decision algorithm which helped the provider and parent identify the families’ priority co-occurring condition and the most feasible strategies that families could implement for their child. After three sessions of the PASS social communication intervention, the non-specialist health workers supported the parent to identify the co-occurring concerns most problematic for the child and family and suggested relevant advice and strategies. Subsequent sessions were then extended to include both core social communication strategies and supportive strategies as appropriate and parents were given handouts to use between sessions for the latter. These sections of the session were delivered as distinct interventions, and the separate “Plus” section was only delivered after three foundational social communication sessions were complete. The distinction between core PASS and the “Plus” elements is clarified in Table 4. In distinction to the collaborative video-feedback facilitation of the core PASS intervention for autistic development, some of the “Plus” modules for co-occurring problems contained practical strategies for the management of day-to-day challenges to caregiving and family functioning (for instance, functional analysis of toileting and sleep difficulties). Written home programs for both components supported families to practice strategies collaboratively set during the session.

**TABLE 4:** List of common components based on the @PracticeWise framework (20).

S.No	Element/ Technique	Source	Practice element label	Definition	Rahman et al. (22)	Divan et al. (29)
1.	Technique	©Practicewise	Accessibility Promotion	Any strategy used to make services convenient and accessible or to proactively enhance treatment participation. This might include: • “appointment reminders” (e.g., telephone, postal, text message or email confirmations and reminders • availability of on-site child care • flexible scheduling, e.g., after-hours scheduling, drop-in appointments • location, e.g., holding sessions at a local school, the family’s home, or other convenient sit • transportation, e.g., to appointments, bus tokens, gas money	Home based delivery, appointment reminders telephone confirmation	As in PASS
2.	Element	©Practicewise	Activity Selection	The identification of specific positive activities in which the child can participate outside of therapy, with the goal of promoting or maintaining involvement in rewarding and enriching experiences.	Collaborative identification of activities for home practice	As in PASS
3.	Element	©Practicewise	Caregiver Coping	Exercises or strategies designed to enhance caregivers’ ability to deal with stressful situations.	No	“Plus” component only: Supportive strategies for parental well-being including referrals for at risk parents
4.	Element	Additional	Communication Skills _Social Communication	Strategies to support the development of joint/shared attention and joint engagement	Video feedback technique to identify and encourage parent behaviors to support social communication during dyadic play	As in PASS
5.	Element	Additional	Communication Skills Language expansion	Strategies to support language expansion	Video feedback technique to identify and support opportunities for language expansion during dyadic play	As in PASS
6.	Element	Additional	Family Engagement	Strategies that are directed beyond the caregiver-child dyad to help wider family members to understand the intervention and support intervention strategies which are being generalized	Initial home visit where family members are supported to increase their understanding of the impairments in autism and the goals of the intervention	As in PASS
7.	Element	©Practicewise	Functional Analysis/Behavior Management	The study of antecedents and consequences impacting a behavior designed to yield a functional understanding of that behavior. This analysis is typically tested through controlled manipulation of antecedents and consequences to verify the formulation.	No	Plus component only: Supporting caregivers to consider the environment around challenging behaviors, and to address contributing factors.
8.	Technique	Additional	Goal Setting-Caregivers	The explicit selection of a therapeutic goal set for the parent for the purpose of working toward achieving that goal. This often involves repeated assessment of the successful approximation of the goal.	Home practice goals collaboratively set with caregiver based on discussions during the session.	As in PASS
9.	Technique	Additional	Handouts	The use of written or illustrated materials to support psycho-education and strategies that caregivers can use to promote specific changes in the child’s behavior	No	Plus component only: Illustrated handouts designed to support understanding and addressing common co-occurring conditions
10.	Technique	Additional	Homework- Review	Review of homework set in the previous therapy session	Home practice goals reviewed at each session	As in PASS
11.	Technique	Additional	Record/diary	Caregiver or non-specialist maintaining records or a diary of child’s behavior or strategies/home program practiced	Written record of key observations from the session along with goals for home practice maintained	As in PASS
12.	Element	©Practicewise	Play Therapy	The use of play as a primary strategy for therapeutic change. This may include the use of play as a strategy for clinical interpretation.	Dyadic play as the basis of the video feedback for social communication intervention	As in PASS
13.	Technique	©Practicewise	Praise- Caregivers	The training of caregivers, teachers, or others involved in the social ecology of the child in the administration of social rewards to promote desired behaviors. This can involve praise, encouragement, affection, or physical proximity.	Validation of strategies used during play and for engagement with home practice is a technique used as a social reward for the caregiver	As in PASS
14.	Element	©Practicewise	Problem Solving	Training in the use of techniques, discussions, or activities designed to bring about solutions to targeted problems, usually with the intention of imparting a skill for how to approach and solve future problems in a similar manner. Includes components such as brainstorming, choosing a solution, and evaluating the results.	Collaborative problem solving to support caregiver to engage with strategies during home practice	As in PASS. Plus component only: Additionally to support adopting strategies to manage co-occurring conditions
15.	Technique	©Practicewise	Psychoeducation - Caregiver	The formal review of information with the caregiver (Socratic or otherwise) about the development of the child’s problem and its relation to a proposed intervention. This often involves an emphasis on the caregiver’s role in either or both. This can include multiple media (e.g., a video about mental health problem).	Initial engagement with the family and caregivers aims at gaining an insight of the families’ understanding of autism and their aspirations of the intervention process.	As in PASS Plus component only: Additionally understanding the family’s interpretation and reasons for co-occurring conditions
16.	Technique	Additional	Relationship/Rapport Building-Caregiver	Strategies in which the primary aim is to increase the quality of the relationship between the therapist and caregiver. Can include active listening and empathy	Reflective video-feedback and collaborative goal setting aims at building a positive therapeutic alliance	As in PASS
17.	Element	©Practicewise	Relaxation	Techniques or exercises designed to induce physiological calming, including muscle relaxation, breathing exercises, imagery, meditation, and similar activities.	No	Plus component only: Supportive relaxation techniques as a first step to help support caregiver well-being
18.	Technique	©Practicewise	Tangible Rewards	The training of parents, teachers, or others involved in the social ecology of the child in the contingent administration of tangible rewards to promote desired behaviors. This can involve tokens, charts, or record keeping, in addition to direct (i.e., first order) reinforcers.	No	Plus component only: Supporting caregivers to consider tangible rewards which can motivate their child while they use techniques to address co-occurring conditions
19.	Technique	Additional	Video feedback	Reviewing caregiver-child interaction videos and providing feedback to the caregiver on the child’s behavior, the impact of the caregivers actions on the child’s behavior and suggestions on alternative ways of interacting.	Reflective video-feedback and collaborative goal setting is the core methodology of this intervention	As in PASS.
						

Divan and colleagues (29) evaluated this “PASS PLUS” intervention in a small effectiveness evaluation, delivered one-on-one by non-specialist health workers in family homes, this time in rural Kolhapur, India. Children with a diagnosis of autism aged 2–9 years were randomly assigned to the PASS PLUS intervention (*n* = 21) or usual care condition (*n* = 19) with stratification for age (<5 or >5) and functional impairment. In practice, the Plus modules were delivered to 79% of the dyads (*n* = 15/19), with 4 families (21%) stating no expressed needs for further support. The most commonly delivered modules in decreasing order were for behavioral challenges (*n* = 12), sensory difficulties (*n* = 7), toileting support (*n* = 5), parental well-being (*n* = 4), sleep problems (*n* = 3) and feeding difficulties (*n* = 2). The study demonstrated high feasibility and intervention sessions met high therapist fidelity thresholds, with 92% of the core PASS procedures meeting fidelity thresholds for mandatory manualized content, and 96% meeting thresholds for quality. This was lower for the supplementary Plus modules, with fidelity thresholds to these manual components being met in 75% of sessions, and 81% meeting quality thresholds. The intervention was reported to be acceptable, and with regards to attendance, 89% of participants in the trial arm received at least the minimum 3 sessions and 68% received the maximum 12. For outcomes, the results showed that there was no evidence that additional delivery of these rather different strategies had a negative effect on the effectiveness of the core PASS video-aided communication intervention; the positive changes in parent synchrony and child initiations in the dyad were replicated as in PASS (22). In addition, PASS PLUS showed some evidence of a trend toward improvement in parental mental wellbeing (as measured by the PHQ-9), (28) but did not lead to significant changes, relative to the control group, in child outcomes such as co-occurring symptom levels (as measured by the Developmental Behavior Checklist, DBC) (30) or autism symptom severity as measured by the Brief Observation of Social Communication Change (BOSCC) (31). A large scale-up effectiveness and cost-effectiveness evaluation of this PASS Plus intervention is currently underway using delivery by frontline workers embedded in the established health system in urban India (32). The Communication-centered Parent mediated treatment for Autism Spectrum Disorders in South Asia (“COMPASS”) trial in this way represents the first large scale effectiveness test of an intervention for autism in a LMIC.

### Common Components

Reviewing the described interventions in the publications along with the knowledge of the intervention, common components across the identified interventions are represented in Tables 4, 5.

**TABLE 5:** List of common components based on the NDBI-Fi framework (11).

S. No.	Components	Brief Description	Rahman et al. (22)	Divan et al. (29)
1.	Face-to-Face (Technique)	• Child and adult facing each other • Child and adult on similar level	The parent is encouraged to take a position which allows them to observe their child’s communication signals	As in PASS
2.	Follow child’s lead (Element)	• Child and adult are both active participants in child-chosen activity	The parent is encouraged to allow the child to choose the toy they would like to play with	As in PASS
3.	Positive affect (Technique)	• Adult uses positive affect • Adult matches affect to child’s sensory needs	The parent is encouraged to be positively involved in the child’s play and validate the child’s choices during the interaction	As in PASS
4.	Modeling language (Element)	• Adult adjusts language to the child’s developmental level	The parent is encouraged to recognize their child’s communication level and match it during play	As in PASS
5.	Responding to communication (Element)	• Adult verbally responds to child’s communication behaviors by repeating, clarifying, or expanding	The parent is encouraged to recognize their child’s communication and initially repeat their outputs. As the child’s communication increases the parent is encouraged to expand the language contingently	As in PASS
6.	Communicative temptations (Elements)	• Adult creates situations to elicit communication from the child followed by a brief period of expectant waiting	As the child and parent build a to and fro play and communication routine, expectant pauses are introduced	As in PASS
7.	Frequency of direct teaching (Technique)	• Adult directs the child to demonstrate new or emerging skills	No	Plus component only: The parent is encouraged to adopt techniques that can help build the child’s adaptive skills for independent functioning such as toileting, eating and sleeping.
8.	Quality of direct teaching (Technique)	• Adult uses high-quality teaching strategies (e.g., clear instructions, when a child is motivated, contingent reinforcement).	No	Plus component only: The parent promotes desired behaviors of the child through strategies such as positive reinforcement, clear instructions, and social stories.

## Discussion

This scoping review set out to identify interventions for young autistic children that have been delivered by non-specialists in low- and middle-income countries (LMIC) with evidence of feasibility, acceptability, and effectiveness. It was hoped that the review would elucidate the intervention elements that have evidence of successful delivery in LMIC by non-specialist providers and offer indications for the future direction in the design, mechanistic evaluation, and delivery of scalable interventions for low resource settings. This review identified only two studies that have evaluated the effectiveness of interventions for young autistic children and their families delivered by non-specialists in LMIC. Both these studies were conducted by teams that included senior authors of this review and both evaluated interventions that were parent-mediated and primarily communication-focused, where the non-specialist had no direct contact with the child beneficiary. There is, therefore, a striking lack of diverse empirical evidence to guide scalable intervention design and delivery within the countries of the world in which most of the world’s autistic children live.

Further clinical research in LMIC is urgently needed to provide evidence that is directly applicable to the key needs of LMIC health systems, to inform task-sharing and non-specialist delivery of autism interventions. This will help reduce inequity and achieve universal coverage within health systems which have an acute paucity of human and economic resources.

This review adopted a narrow definition of non-specialist providers to accurately reflect the most abundant workforce resource available to task-sharing approaches within LMIC: frontline community health workers, community volunteers or parent/disability champions. This focus allows the results to be more directly applicable to these settings. Other reviews (33) have investigated “non-specialist” delivery but adopted a wider definition of practitioners who are not relevant to the many LMIC settings that we are discussing. Frontline community health workers provide advice and support around issues such as maternity care, nutrition, immunization, child growth and development. They are often educated through high school but are not necessarily graduates; they have no formal healthcare or child development training but receive focused training around specific issues. They often live within the communities they serve and therefore likely to be trusted by the community. These frontline workers are an invaluable and flexible resource that can be trained, supervised, and incentivized to deliver interventions to children with neurodevelopmental disabilities and their families.

In this review we used two pre-established frameworks to classify intervention components, within the two interventions identified. We showcase that non-specialists – supported by a carefully established and implemented supervisory framework - can deliver a range of intervention elements and techniques to parents of young autistic children, with preliminary evidence of feasibility, acceptability, and effectiveness. Six elements from the @Practisewise framework (20) and four elements from the NDBI framework (17) along with three additional elements, were shown to be successfully delivered in this way. These included supporting parents to understand the communication development of their autistic child and to use strategies (e.g., language expansion, communication temptations) to support communication and language development. Similarly, we identified four techniques from the @Practisewise framework (20) and four from the NDBI framework (17) along with five additional techniques, including collaborative goal setting, parental psychoeducation, and the use of positive social or tangible rewards. It is important to note that we have not identified any tests of whether non-specialists could deliver intervention components directly with the autistic child or whether they could deliver interventions targeting other developmental domains or more complex co-occurring conditions, such as anxiety. These are important foci for future work. Another important clarification is that we are not proposing that the elements and techniques that we have identified are *active ingredients.* This would require in-depth mechanistic studies of each element and their interactions with each other and with outcomes (34, 35). A priority for future research should be to identify such active ingredients but also to evaluate the extent to which those ingredients can be delivered by non-specialists. Our conclusions on components must be tempered by the fact that they are based on a very small number of studies testing two interlinked interventions. However, this analysis can help clinicians and researchers to select from these evidence-based intervention components which have proved to be feasible for non-specialist delivery to inform scalable autism interventions implemented through the task-sharing approach.

An important question concerns the equivalence of task-sharing approaches in comparison to specialist-delivered approaches. The task-sharing approach has evidence of feasibility and acceptability within the context of adult mental health across diverse low resource settings (36–39) and some supportive evidence of equivalence. For example, a head-to-head comparison within a depression treatment study (40) found that a simpler psychological treatment delivered using the task-sharing approach had the same effect as a costlier intervention delivered by specialists. This suggests that well-designed training and supervision cascades led by highly qualified and expensive experts can support less qualified individuals to deliver complex interventions with similar effectiveness but to more people and at a lower cost. We do not have any directly comparable results on task-sharing versus specialist delivery for autism interventions and this is an important focus for future research if we are to ensure health equity across populations.

**FIGURE 2:**
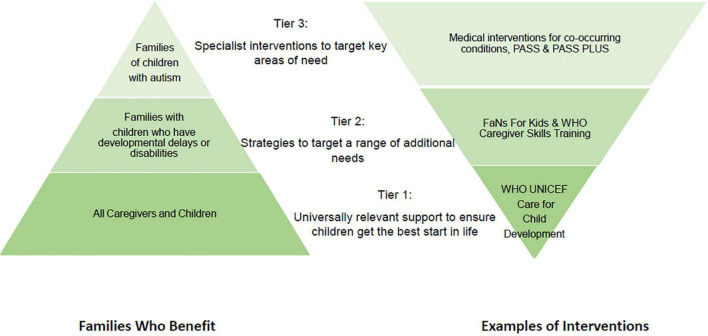
Stepped care approach to supporting children based on the WHO Nurturing Care Framework.

The Lancet commission on the Future of Care and Clinical Research for Autism (41) recognizes the prohibitive costs and lack of access to evidence-based interventions in most of the world. The authors recommend a stepped care personalized approach to care, (5, 42) and we would suggest that interventions containing the common components identified here could be evaluated as WHO tier 3 interventions for delivery in low resource settings, allowing more complex cases to be triaged up to specialist care. As part of this stepped care approach, non-specialist delivery could be used to widen access to support, potentially benefiting many additional children, including those with other neurodevelopmental disabilities (Figure 2).

That said, even these common components delivered in one setting may have challenges in others, which may include the professional and personal workload of the frontline worker. Additionally, there will need to be contextual adaptations based on community perceptions and needs along with structural systemic changes to support career progression of frontline workers who may be trained to deliver such complex interventions.

In addition to our two identified papers, there were studies which did not meet our inclusion criteria, but which may also inform decisions around non-specialist delivery. We have included a couple of informative examples here. Hamdani and colleagues (43) conducted a large (*n* = 540) single-blinded, cluster randomized controlled trial in rural Rawalpindi, Pakistan. The trial evaluated the “FaNs for Kids” intervention compared to enhanced treatment-as-usual for parents of children aged 2–12 with a range of developmental delays and disabilities. The sample included children with communication disabilities but not formally diagnosed autism and, for this reason, did not meet the inclusion criteria of our search. FaNs for Kids was a group-based, psycho-education intervention adapted from the WHO Mental Health Gap Intervention Guidelines (mhGap-IG). The programme targeted child development and functioning (communication, socioemotional development, co-occurring conditions, and motor difficulties), as well as caregiver psychological well-being, and was delivered by “family champions,” volunteers with family experience of disability, under the supervision of master trainers. The intervention was technologically assisted: caregivers accessed training videos on a tablet device and then met in nine group-based sessions to discuss different scenarios and build management plans around their child’s needs. The study found that FaNs for Kids resulted in improved health-related quality of life in caregivers, but there were no significant improvements on child outcomes, relative to the control group.

Through this study, Hamdani and colleagues demonstrated that technology can be an effective medium to deliver psychosocial interventional components to parents within low resourced settings. This highlights the potential held by technology-assisted interventions to scale-up intervention delivery in such settings which because of the standardized messaging can help maintain program fidelity as well as ensure intervention dosage by non-specialists. In PASS (22) and PASS Plus (29) non-specialists also make use of digital technology, through the use of video feedback techniques. Within an ongoing trial of the PASS Plus intervention delivered by frontline health workers in Delhi, India, digital technology is being harnessed further to overcome the challenges presented by the COVID-19 pandemic and enable ongoing training and supervision of the workforce and intervention delivery to families using video-conferencing and telephonic technology (44). Lakhera and colleagues evaluated the feasibility of this digitally assisted delivery of PASS Plus and the acceptability of virtually adapted sessions to 50 families of children with autism, 34 families who transitioned from receiving home based delivery of PASS Plus, and 16 families who received virtual delivery only. Though there were several barriers to virtual delivery of sessions, including a family preference for home-based delivery, the lack of access to the internet and lack of digital literacy, most families and non-specialist providers adapted successfully to the remote delivery of the intervention. Such innovations of technology – if integrated within the existing health care system – may help to leap-frog barriers to scaling up service delivery.

A second programme of research that did not meet our inclusion criteria but that may also inform decisions around non-specialist delivery is the World Health Organization Caregiver Skills Training programme (CST) (45). The CST is a trans-diagnostic, family centered intervention designed to be delivered by non-specialists and to fit within Tier 2 of the World Health Organisation (WHO) stepped-care model (i.e., targeted support for children with identified developmental needs; see Figure 2) and thereby bridge the treatment gap for children in low resource settings (46). The CST combines group sessions for caregivers with home visits to tailor the intervention to each family and set individual goals for each child (47). The intervention is informed by a range of approaches including psycho-education, social communication interventions, functional analysis, positive parenting, and self-care methods with a strong foundation in the WHO-UNICEF Nurturing Care Framework (48). At the point of this scoping review, there were no published systematic evaluations of effectiveness using non-specialist providers. However, feasibility and acceptability studies have been conducted (49, 50), and we have been informed of evaluations of non-specialist delivery under review. The intervention has been pre-piloted through specialist workers in Italy and is now being field tested, including with non-specialist providers, in more than 30 countries around the world (50).

The strengths of this publication are that we have conducted a rigorous scoping review and have used two frameworks to classify intervention components allowing us to identify key techniques and strategies that non-specialists have delivered with evidence of effectiveness in three settings of urban and rural South Asia to families of young children with autism. A further strength concerns the explicit definition of non-specialist adopted in this review. However, this process has highlighted the acute lack of empirical data that is directly applicable to the needs of LMIC health systems, and is an urgent indication for more relevant research to support families of children with autism in the most underserved populations of the world.

## Data Availability Statement

The original contributions presented in this study are included in the article/supplementary material, further inquiries can be directed to the corresponding author.

## Author Contributions

GD and KL conceived the idea for the research manuscript and defined the inclusion criteria for the search. LN, CG, AK, CB, and SL screened abstracts and full-text manuscript based on the search criteria on Rayyan software with support from GD and KL. LN, CG, CB, AK, KL, and GD drafted the first version of the manuscript and contributed to subsequent versions. All authors read and approved the final manuscript.

## Conflict of Interest

LN, AK, SL, and GD were employed by the not-for-profit Sangath. GD has led on the development and evaluation of the PASS and PASS Plus interventions and has received funding from Autism Speaks US and Grand Challenges Canada for this work. KL was also supported by an Autism Speaks grant for work on these interventions. The remaining authors declare that the research was conducted in the absence of any commercial or financial relationships that could be construed as a potential conflict of interest.

## Publisher’s Note

All claims expressed in this article are solely those of the authors and do not necessarily represent those of their affiliated organizations, or those of the publisher, the editors and the reviewers. Any product that may be evaluated in this article, or claim that may be made by its manufacturer, is not guaranteed or endorsed by the publisher.

## References

[1] Baio J Wiggins L Christensen DL Maenner MJ Daniels J Warren Z Prevalence of autism spectrum disorder among children aged 8 years—autism and developmental disabilities monitoring network, 11 sites, United States, 2014. *MMWR Surveill Summ.* (2018) 67:1. 10.15585/mmwr.ss6706a1 29701730 PMC5919599

[2] Elsabbagh M Divan G Koh YJ Kim YS Kauchali S Marcín C Global prevalence of autism and other pervasive developmental disorders. *Autism Res.* (2012) 5:160–79. 10.1002/aur.239 22495912 PMC3763210

[3] Perera H Wijewardena K Aluthwelage R. Screening of 18–24-month-old children for autism in a semi-urban community in Sri Lanka. *J Trop Pediatr.* (2009) 55:402–5. 10.1093/tropej/fmp031 19401407

[4] Arora NK Nair MK Gulati S Deshmukh V Mohapatra A Mishra D Neurodevelopmental disorders in children aged 2–9 years: population-based burden estimates across five regions in India. *PLoS Med.* (2018) 15:e1002615. 10.1371/journal.pmed.1002615 30040859 PMC6057634

[5] Divan G Bhavnani S Leadbitter K Ellis C Dasgupta J Abubakar A Annual research review: achieving universal health coverage for young children with autism spectrum disorder in low-and middle-income countries: a review of reviews. *J Child Psychol Psychiatry.* (2021) 62:514–35. 10.1111/jcpp.13404 33905120

[6] World Health Organisation. *Mental Health Atlas 2020.* Geneva: World Health Organisation (2021).

[7] United Nations. *Goal 3, The Sustainable Development Goals Report.* (2021). Available online at: https://sdgs.un.org/goals/goal3 (accessed February 28, 2022).

[8] Raviola G Naslund JA Smith SL Patel V. Innovative models in mental health delivery systems: task sharing care with non-specialist providers to close the mental health treatment gap. *Curr Psychiatry Rep.* (2019) 21:1–3. 10.1007/s11920-019-1028-x 31041554

[9] Singla DR Kohrt BA Murray LK Anand A Chorpita BF Patel V. Psychological treatments for the world: lessons from low-and middle-income countries. *Annu Rev Clin Psychol.* (2017) 13:149–81. 10.1146/annurev-clinpsy-032816-045217 28482687 PMC5506549

[10] Van Ginneken N Tharyan P Lewin S Rao GN Meera SM Pian J Non-specialist health worker interventions for the care of mental, neurological and substance-abuse disorders in low-and middle-income countries. *Cochr Database Syst Rev.* (2013) 2013:CD009149. 10.1002/14651858.CD009149.pub2 24249541

[11] Mane Abhay B Khandekar Sanjay V. Strengthening primary health care through Asha workers: a novel approach in India. *Primary Health Care.* (2014) 4:2167–1079.

[12] Shrestha R Barbaro J Dissanayake C. Changes in knowledge on the signs of autism in young children (11–30 months) among female community health volunteers in Nepal. *J Autism Dev Disord.* (2022) 52:219–39. 10.1007/s10803-021-04944-7 33709379

[13] Aseyo RE Mumma J Scott K Nelima D Davis E Baker KK Realities and experiences of community health volunteers as agents for behaviour change: evidence from an informal urban settlement in Kisumu, Kenya. *Hum Resour Health.* (2018) 16:1–2. 10.1186/s12960-018-0318-4 30286763 PMC6172748

[14] Naveed S Waqas A Amray AN Memon RI Javed N Tahir MA Implementation and effectiveness of non-specialist mediated interventions for children with autism spectrum disorder: a systematic review and meta-analysis. *PLoS One.* (2019) 14:e0224362. 10.1371/journal.pone.0224362 31703073 PMC6839885

[15] Nair MK Russell PS George B Prasanna GL Bhaskaran D Leena ML CDC Kerala 9: effectiveness of low intensity home based early intervention for autism spectrum disorder in India. *Indian J Pediatr.* (2014) 81:115–9. 10.1007/s12098-014-1474-8 25141828

[16] Chorpita BF Daleiden EL Weisz JR. Identifying and selecting the common elements of evidence based interventions: a distillation and matching model. *Ment Health Servic Res.* (2005) 7:5–20. 10.1007/s11020-005-1962-6 15832690

[17] Frost KM Brian J Gengoux GW Hardan A Rieth SR Stahmer A Identifying and measuring the common elements of naturalistic developmental behavioral interventions for autism spectrum disorder: development of the NDBI-Fi. *Autism.* (2020) 24:2285–97. 10.1177/1362361320944011 32731748 PMC7541530

[18] Levac D Colquhoun H O’Brien KK. Scoping studies: advancing the methodology. *Implement Sci.* (2010) 5:1–9. 20854677 10.1186/1748-5908-5-69 PMC2954944

[19] Ouzzani M Hammady H Fedorowicz Z Elmagarmid A. Rayyan—a web and mobile app for systematic reviews. *Syst Rev.* (2016) 5:1–0. 10.1186/s13643-016-0384-4 27919275 PMC5139140

[20] Chorpita BF Daleiden EL. Mapping evidence-based treatments for children and adolescents: application of the distillation and matching model to 615 treatments from 322 randomized trials. *J Consult Clin Psychol.* (2009) 77:566–79. 10.1037/a0014565 19485596

[21] PracticeWise. *Psychosocial and Combined Treatments Coding Manual.* Satellite Beach, FL: PracticeWise (2012).

[22] Rahman A Divan G Hamdani SU Vajaratkar V Taylor C Leadbitter K Effectiveness of the parent-mediated intervention for children with autism spectrum disorder in south Asia in India and Pakistan (PASS): a randomised controlled trial. *Lancet Psychiatry.* (2016) 3:128–36. 10.1016/S2215-0366(15)00388-0 26704571

[23] Divan G Hamdani SU Vajartkar V Minhas A Taylor C Aldred C Adapting an evidence-based intervention for autism spectrum disorder for scaling up in resource-constrained settings: the development of the PASS intervention in South Asia. *Glob Health Action.* (2015) 8:27278. 10.3402/gha.v8.27278 26243710 PMC4524889

[24] Aldred C Green J Adams C. A new social communication intervention for children with autism: pilot randomised controlled treatment study suggesting effectiveness. *J Child Psychol Psychiatry.* (2004) 45:1420–30. 10.1111/j.1469-7610.2004.00848.x 15482502

[25] Sparrow SS Cicchetti DV Balla DA. *Vineland Adaptive Behavior Scales.* 2nd ed. Livonia, MN: Pearson Assessment (2006).

[26] Fenson L Marchman VA Thal DJ Dale PS Reznick JS. *MacArthur-Bates Communicative Development Inventories.* 2nd ed. Baltimore, MD: Paul H. Brookes (2007).

[27] Wetherby AM Prizant BM. *Communication and Symbolic Behavior Scales: Developmental Profile.* Baltimore, MD: Paul H. Brookes (2002).

[28] Kroenke K Spitzer RL Williams JB. The PHQ-9: validity of a brief depression severity measure. *J Gen Intern Med.* (2001) 16:606–13. 10.1046/j.1525-1497.2001.016009606.x 11556941 PMC1495268

[29] Divan G Vajaratkar V Cardozo P Huzurbazar S Verma M Howarth E The feasibility and effectiveness of PASS plus, a lay health worker delivered comprehensive intervention for autism spectrum disorders: pilot RCT in a rural low and middle income country setting. *Autism Res.* (2019) 12:328–39. 10.1002/aur.1978 30095230

[30] Einfeld SL Tonge BJ. *Manual for the Developmental Behaviour Checklist (Primary Carer Version).* Melbourne, VIC: Monash University Centre for Developmental Psychiatry (1994).

[31] Grzadzinski R Carr T Colombi C McGuire K Dufek S Pickles A Measuring changes in social communication behaviors: preliminary development of the brief observation of social communication change (BOSCC). *J Autism Dev Disord.* (2016) 46:2464–79. 10.1007/s10803-016-2782-9 27062034

[32] Sangath. *Communication-Centred Parent-Mediated Treatment for Autism Spectrum Disorder in South Asia (COMPASS).* Delhi: Sangath (2018).

[33] Reichow B Servili C Yasamy MT Barbui C Saxena S. Non-specialist psychosocial interventions for children and adolescents with intellectual disability or lower-functioning autism spectrum disorders: a systematic review. *PLoS Med.* (2013) 10:e1001572. 10.1371/journal.pmed.1001572 24358029 PMC3866092

[34] Green J Garg S. Annual research review: the state of autism intervention science: progress, target psychological and biological mechanisms and future prospects. *J Child Psychol Psychiatry.* (2018) 59:424–43. 10.1111/jcpp.12892 29574740

[35] Gulsrud AC Hellemann G Shire S Kasari C. Isolating active ingredients in a parent-mediated social communication intervention for toddlers with autism spectrum disorder. *J Child Psychol Psychiatry.* (2016) 57:606–13. 10.1111/jcpp.12481 26525461 PMC8320675

[36] Ali BS Rahbar MH Naeem S Gul A Mubeen S Iqbal A. The effectiveness of counseling on anxiety and depression by minimally trained counselors: a randomized controlled trial. *Am J Psychother.* (2003) 57:324–36. 10.1176/appi.psychotherapy.2003.57.3.324 12961817

[37] Chatterjee S Chowdhary N Pednekar S Cohen A Andrew G Andrew G Integrating evidence-based treatments for common mental disorders in routine primary care: feasibility and acceptability of the MANAS intervention in Goa, India. *World Psychiatry.* (2008) 7:39–46. 10.1002/j.2051-5545.2008.tb00151.x 18458786 PMC2359726

[38] Chibanda D Mesu P Kajawu L Cowan F Araya R Abas MA. Problem-solving therapy for depression and common mental disorders in Zimbabwe: piloting a task-shifting primary mental health care intervention in a population with a high prevalence of people living with HIV. *BMC Public Health.* (2011) 11:828. 10.1186/1471-2458-11-828 22029430 PMC3210104

[39] Rahman A Malik A Sikander S Roberts C Creed F. Cognitive behaviour therapy-based intervention by community health workers for mothers with depression and their infants in rural Pakistan: a cluster-randomised controlled trial. *Lancet.* (2008) 372:902–9. 10.1016/S0140-6736(08)61400-2 18790313 PMC2603063

[40] Richards DA Ekers D McMillan D Taylor RS Byford S Warren FC Cost and outcome of behavioural activation versus cognitive behavioural therapy for depression (COBRA): a randomised, controlled, non-inferiority trial. *Lancet.* (2016) 388:871–80. 10.1016/S0140-6736(16)31140-0 27461440 PMC5007415

[41] Lord C Charman T Havdahl A Carbone P Anagnostou E Boyd B The lancet commission on the future of care and clinical research in autism. *Lancet.* (2022) 399:271–334. 10.1016/S0140-6736(21)01541-5 34883054

[42] Green J Leadbitter K Ainsworth J Bucci S. An integrated early care pathway for autism. *Lancet Child Adolesc Health.* (2022) 6:335–44. 10.1016/S2352-4642(22)00037-2 35303486

[43] Hamdani SU Suleman N Akhtar P Nazir H Masood A Tariq M Effectiveness of a technology-assisted, family volunteers delivered, brief, multicomponent parents’ skills training intervention for children with developmental disorders in rural Pakistan: a cluster randomized controlled trial. *Int J Ment Health Syst.* (2021) 15:1–7. 10.1186/s13033-021-00476-w 34059074 PMC8165981

[44] Lakhera S Roy SG Laithani N Sangwan P Azar Z Menon S Adaptation of a parent mediated communication intervention for autism for virtual delivery, in India. In: *Proceedings of the International Society for Autism Research*. (2021).

[45] Salomone E Pacione L Shire S Brown FL Reichow B Servili C. Development of the WHO caregiver skills training program for developmental disorders or delays. *Front Psychiatry.* (2019) 10:769. 10.3389/fpsyt.2019.00769 31780960 PMC6859468

[46] World Health Organisation. *WHO Caregivers Skills Training for Families of Children With Developmental Delays and Disorders.* (2022). Available online at: https://www.who.int/teams/mental-health-and-substance-use/treatment-care/who-caregivers-skills-training-for-families-of-children-with-developmental-delays-and-disorders (accessed March 3, 2022).

[47] Tekola B Girma F Kinfe M Abdurahman R Tesfaye M Yenus Z Adapting and pre-testing the World Health Organization’s caregiver skills training programme for autism and other developmental disorders in a very low-resource setting: findings from Ethiopia. *Autism.* (2020) 24:51–63. 10.1177/1362361319848532 31094208 PMC6927066

[48] World Health Organization, United Nations Children’s Fund, World Bank Group. *Nurturing Care for Early Childhood Development: a Framework for Helping Children Survive and Thrive to Transform Health and Human Potential*. Geneva: World Health Organisation (2018).

[49] Sengupta K Shah H Ghosh S Sanghvi D Mahadik S Dani A World Health Organisation-caregiver skills training (WHO-CST) program: feasibility of delivery by non-specialist providers in real-world urban settings in India. *J Autism Dev Disord.* (2021) 51:1–8. 10.1007/s10803-021-05367-0 34853959

[50] Salomone E Ferrante C Salandin A Ferrara F Torchio E Foletti G Acceptability and feasibility of the World Health Organization’s caregiver skills training implemented in the Italian national health system. *Autism.* (2021) 25:13623613211035228. 10.1177/13623613211035228 34362266

